# Intestinal helminth *Schyzocotyle acheilognathi* Yamaguti, 1934 infection ameliorate lipid metabolism of grass carp (*Ctenopharyngodon idella*) through immune and gut microbiota regulation

**DOI:** 10.3389/fmicb.2025.1538919

**Published:** 2025-07-10

**Authors:** Xiaoao Yang, Denghui Zhu, Wenxiang Li, Peipei Fu

**Affiliations:** ^1^National Engineering Research Center of Marine Facilities Aquaculture, Marine Science and Technology College, Zhejiang Ocean University, Zhoushan, China; ^2^National Engineering Laboratory of Marine Germplasm Resources Exploration and Utilization, Marine Science and Technology College, Zhejiang Ocean University, Zhoushan, China; ^3^Key Laboratory of Breeding Biotechnology and Sustainable Aquaculture (CAS), Institute of Hydrobiology, Chinese Academy of Sciences, Wuhan, China

**Keywords:** *Schyzocotyle acheilognathi*, *Ctenopharyngodon idella*, high-fat diet, lipid metabolism, gut microbiota

## Abstract

Fats have been widely applied in aquaculture to promote growth performance and substitute partial protein in fish feeds. However, excessive dietary fat levels induce metabolic disorders harming the health of cultured fish. Helminth infection in mammals was inversely correlated with metabolic syndrome, but its effect in aquatic animals is unknown yet. Here, we evaluated the impacts of *Schyzocotyle acheilognathi* infection on lipid metabolism of grass carp fed with high-fat diet (HFD). By comparison with the uninfected grass carp, helminth infection significantly increased the concentration of high-density lipoprotein (HDL) and condition factor (CF), and significantly decreased the concentration of low-density lipoprotein (LDL), the activity of AST, perimeter ratio (PR) and the thickness of muscularis mucosa (MM). Helminth infection also significantly lowered the lipid accumulation in liver, which may attribute to the significant up-regulated expression levels of apolipoprotein E (*ApoE*) and down-regulated expression of peroxisome proliferator-activated receptor-gamma (*PPAR-*γ) and lipoprotein lipase (*LPL*). Meanwhile in the grass carp infected by tapeworm, there was significant down-regulated expression of pro-inflammatory genes, interleukin-1beta (*IL-1*β) and tumor necrosis factor-alpha (*TNF-*α), and significant up-regulated expression of anti-inflammatory genes, transforming growth factor-beta 1 (*TGF-*β*1*) and interleukin-10 (*IL-10*). 16S rDNA sequencing results showed that helminth infection didn’t affect the α diversity of the intestinal microbiota, but increased the relative abundance of *Cetobacterium*, and significantly changed the structure of intestinal microbiota by PERMANOVA analysis. Correlation analysis showed the relative abundance of *Cetobacterium* was significant positively correlated with the helminth infection in grass carp fed HFD. PICRUST2 analysis indicated that several lipid metabolism-related pathways were significantly altered after helminth infection. Consequently, the above results indicated that tapeworm infection could ameliorate abnormal lipid metabolism through immune and gut microbiota regulation.

## Introduction

Fat is one of the most important sources of nutrition for aquatic organisms, providing essential fatty acids, cholesterol, phospholipids and fat-soluble vitamins needed for normal growth, development and maintenance of the health of farmed fish (Malcolm, 2011). Previous studies indicated that increasing dietary fat content within proper range (5% for herbivorous fish, 8% for omnivorous fish and 10% for carnivorous fish) can boost growth performance, improve reproductive characteristics, exert a protein-sparing effect and decreased feed and production expenses (Boujard, 2004; Chou and Shiau, 1996; Li et al., 2012; Zhang et al., 2017). Thus, high fat diet (HFD) has been extensively utilized in intensive aquaculture. However, long-term excessively fat in the diet induced many adverse implications on farmed fish, increasing fat accumulation in liver, stimulation endoplasmic reticulum stress, impairment the intestinal mucosal barrier, triggering inflammatory responses, imbalance of microbiota and disturbance of metabolism (Cao et al., 2019; Jia et al., 2020a; Jia et al., 2020b; Jin et al., 2019; Tao et al., 2018; Yin et al., 2021; Yu et al., 2020). Therefore, addressing the metabolic imbalance and physiological disturbances induced by HFD will greatly advance the sustainable and healthy development of the aquaculture industry.

Parasitic helminths, mostly considered detrimental to host health, are common macrobiota in gastrointestinal (GI) tract of vertebrates (Peachey et al., 2017). However, several application researches of helminth in some chronic inflammation-related diseases demonstrated the positive effects of parasites on the health of host in recent years (Sobotkova et al., 2019). Infection with the nematode *Heligmosomoides polygyrus* has preventive and therapeutic roles on obesity caused by HFD in mice (Shimokawa et al., 2019). Transient infection with *Nippostrongylus brasiliensis* (nematode) in mice long-lasting improved insulin sensitivity and decreased adipose tissue mass in HFD obese mice (Wu et al., 2011; Yang et al., 2013). Chronic infection with the digenean *Schistosoma mansoni* or *S. mansoni-*soluble egg antigens (SEAs), a mixture of helminth-derived molecules, both improved insulin sensitivity and glucose homeostasis (Hussaarts et al., 2015). The above studies suggested that helminth infection or products derived from helminths promoted metabolic benefit for health of mammal hosts (Guigas and Molofsky, 2015). However, it remains unclear whether the protective effects of helminth against metabolic diseases also exists in aquatic animals.

Grass carp (*Ctenopharyngodon idella*) is one of the most important economic freshwater aquaculture species in China, and its production reached 5.9 million tons, accounting for 21.8% of the total annual production of freshwater farmed fish in 2023, according to the China Fishery Statistical Yearbook (Liu, 2023). Previous studies have suggested that a diet containing 4% lipids optimizes growth performance, feed efficiency, and the protein-sparing effect in juvenile grass carp (Du et al., 2005), while excess dietary fat level induced growth performance reduction, lipid deposition in liver, muscle and mesenteric tissue, damage of intestinal mucosal barrier and imbalance of intestinal microbiota (Du et al., 2006; Liu et al., 2022; Liu et al., 2023; Tang et al., 2019). Therefore, alleviating the detrimental impacts caused by HFD is crucial for the health of grass carp.

*Schyzocotyle acheilognathi* (*syn. Bothriocephalus acheilognathi*) is a common helminth species harboring in foregut of grass carp (Kuchta et al., 2018; Liao and Shi, 1956). In our previous study, *S. acheilognathi* infection altered the composition of gut microbiota (Fu et al., 2022). Studies in mammals have shown that helminth improved metabolic diseases through gut microbiota. The composition and diversity of the intestinal microbiota in vertebrates are always altered by helminth infection (Peachey et al., 2017). A limited number of consistent changes in the composition of the host’s gut microbiota have been repeatedly noted in animals infected with helminth (Peachey et al., 2017), called helminth-modified microbiota, which affects host immunity or metabolic capacity (Brosschot and Reynolds, 2018). The *H. polygyrus* infection protected against HFD-induced obesity by altering the composition of the gut microbiota, which led to an increase in norepinephrine (NE) concentration (Shimokawa et al., 2019), or elevated levels of short chain fatty acids (SCFAs) (Su et al., 2020). Thus, this study aims to elucidate the roles and underlying mechanisms of the protective effects of helminths against metabolic diseases in aquatic animals, using grass carp infected with the helminth *S. acheilognathi*, with the goal of offering novel strategies for the treatment of metabolic diseases in aquatic species.

## Materials and methods

### Experimental animals

The fry of grass carp (11 ± 1 cm, 11.4 ± 0.5 g) was purchased from an aquaculture pond in Jiangmen, Guangdong Province, where the prevalence and intensity of *S. acheilognathi* in grass carp were 40% and 3.2, respectively in previous survey. Grass carp were kept temporarily for 3 days prior to the formal experiment.

### Feeding and samples collection

Grass carp were randomly divided into four buckets, fed with a normal diet (ND) with 5% fat content or a high-fat diet (HFD) with 10% fat content (Huaian Tongwei Feed Co., Ltd., China; Table 1) to apparent satiation twice daily (10: 00, 17: 00 oor ersity of se mationertebradomesticated in 100 L plastic buckets under a natural photoperiod (12 h light: 12 h dark), and kept at a water depth of 60 cm and a water temperature of 29°C. Following a 4-week feeding trial, the grass carp (*n* = 63) were anesthetized with eugenol (0.2 mL/L) before sampling.

**TABLE 1:** Composition and nutrient level of feed (air-dry basis).

Ingredients (g/kg)	Con	HFD
Fish meal	120	120
Soybean meal	240	240
Rapeseed meal	300	300
Wheat meal	250	250
Soybean oil	8	50
Ca(H_2_PO_4_)2	20	20
Vitamin mixture^a^	10	10
Mineral mixture^b^	10	10
Cellulose	42	0
**Proximate composition (%)**		
Crude protein	30.0	30.0
Crude fat	5.0	10.0
Ash	15.0	14.8
Moisture	12.5	12.4

^a^Vitamin premix (IU or mg/kg): vitamin A, 65,000 IU; vitamin D_3_, 45,000 IU; vitamin C, 1,200 mg; vitamin E, 250 mg; vitamin K3, 50 mg; vitamin B1, 125 mg; vitamin B2, 150 mg, vitamin B6, 150 mg; vitamin B12, 0.25 mg; niacinamide 500 mg; pantothenate 400 mg; inositol 750 mg; folic acid 250 mg; biotin 0.8 mg. Cellulose was used as a carrier.

^b^Mineral premix (mg/kg): MnSO_4_⋅H_2_O 0.8; MgSO_4_ 100; ZnSO_4_⋅7H_2_O 3.53; CuSO_4_⋅5H_2_O 0.40; CaCO_3_ 150; NaCl 10; KCl 100; AlCl_3_⋅6H_2_O 0.6; KH_2_PO_4_ 220; Ca(H_2_PO_4_)_2_⋅H_2_O 300; CoCl_2_⋅6H_2_O 0.60; KIO_3_⋅6H_2_O, 0.03; ferric citrate 25.

The growth indices of the grass carp were measured, and blood samples were drawn from the caudal vein. Liver samples for gene expression analysis and oil red O staining were stored at −80°C and fixed in 4% paraformaldehyde (PFA) solution, respectively. The intestine was aseptically excised from visceral organs. The foregut and midgut were used for parasites checking under a stereomicroscope (Leica, Germany) to determine whether the host was infected with *S. acheilognathi*. Hindgut contents, intended for 16S rDNA high-throughput sequencing, were stored at −80°C for DNA extraction, and hindgut samples for hematoxylin and eosin (HE) staining were fixed in 4% PFA.

Based on the helminth infection status and the fat content in the feed, the samples were divided into four groups: ND (5% normal fat diet with no *S. acheilognathi* infection), ND + SA (5% normal fat diet with *S. acheilognathi* infection), HFD (10% high fat diet with no *S. acheilognathi* infection) and HFD + SA (10% high fat diet with *S. acheilognathi* infection). After dissection, it was recorded that the mean intensity of *S. acheilognathi* in grass carp was 3.9 (2–7).

### Growth indices

The standard length (L) and body weight (W) of the grass carp were measured to calculate the condition factor (CF). The liver weight (Wl), viscera weight (Wv), and mesenteric fat weight (Wm) were measured individually to determine the hepatosomatic index (HSI), mesenteric fat index (MFI), and visceral index (VSI), respectively. The formulas for calculation of these indicators were shown as follows: CF = W/(L^3^) × 100%; HSI = Wl/W × 100%; VSI = Wv/W × 100%; MFI = Wm/W × 100%.

### Serum biochemical index analysis

Blood samples were centrifuged at 3,000 rpm for 10 min at 4°C, after which the supernatant serum was carefully collected and stored at −80°C until used. The activity of alanine aminotransferase (ALT), aspartate aminotransferase (AST), as well as the concentration of high-density lipoprotein cholesterol (HDL), low-density lipoprotein cholesterol (LDL), total triglycerides (TG), and total cholesterol (TC) in serum were measured through a biochemical analyzer, using commercially available reagent kits (Seville Biotech Co., Ltd., Wuhan, China).

### Histological analysis of intestine

Fresh hindgut tissues were immersed in 4% PFA solution for 48 h, and then processed routinely. These tissues were sectioned into 6 μm slices and stained with hematoxylin and eosin for examination under a light-microscopic (Zeiss, Germany). Photographs were analyzed using software ImageJ (National Institutes of Health, Bethesda, MD, United States). The thickness of muscularis mucosa (MM), the height of microvilli (MV) and perimeter ratio (PR) were measured, respectively. The PR was calculated using the following formulas: PR = the internal perimeter (IP) of the intestine lumen (villi and mucosal folding length)/the external perimeter (EP) of the intestine.

### Oil red O staining

Liver samples from grass carp were excised and preserved in 4% PFA for 24 h. Subsequently, the tissues were trimmed to ensure smoothness and then dehydrated in 30% sucrose solution at 4°C for 24 h. Once the surface liquid of the liver tissue was evaporated, it was embedded in OCT embedding agent (SAKURA, United States). After freezing at −80°C, the tissue was then sectioned into 8 μm thick slices using a cryostat microtome (Leica, Germany) and stained with oil red O. Photomicrographs were captured using a light microscope (Zeiss, Germany). A total of six random fields of view were chosen from each sample, and the areas of lipid droplets were calculated using software ImageJ.

### qPCR

RNAiso Plus Reagent (Takara Bio Inc., Beijing, China) was used to extract total RNA. After being treated with RNase free DNase I (Promega, Wisconsin, United States), single-strand cDNA was synthesized using One−Step gDNA Remover kit (Servicebio, Wuhan, China). qPCR was carried out in a CFX96™ Real Time Detection System (BIO-RAD Bio Inc., Shanghai, China) using TB Green^®^ Premix Ex Taq™ (Beijing, China). Gene-specific primers (Table 2) were used to amplify the target gene fragments. The β*-actin* gene of grass carp (Accession No. M25013.1) served as the reference gene. The qPCR cycle conditions were as follows: an initial denaturation at 95°C for 30 s, followed by 40 cycles of denaturation at 95°C for 5 s, annealing at 60°C for 34 s, and a final Melt Curve analysis. The Ct method (2^–ΔΔCT^) (Livak and Schmittgen, 2001) was used to determine the relative expression levels of immune and lipid metabolic related genes in liver of grass carp.

**TABLE 2:** Primers used for qPCR.

Gene	Sequences of primers	Accession no.
β*-actin*	5′*-*AAGGCCAACAGGGAAAAGAT*-*3′	XM_051889040.1
	5′*-*CATCACCAGAGTCCATCACG*-*3′	
*TGF-*β*1*	5′*-*GTGACGCCAGCATTGTATCTA*-*3′	XM_051877578.1
	5′*-*GTCAGCGTTGCGGAATTTATC*-*3′	
*IL-l*β	5′*-*CCAAGTGCCACCCCGAATGC*-*3′	XM_051908147.1
	5′*-*AGGGGAAGAACCATCCGACTCG*-*3′	
*TNF-a*	5′*-*TGATGGTGTCGAGGAGGAAGGC*-*3′	XM_051871730.1
	5′*-*TTGAGCGTGAAGCAGACAGCAG*-*3′	
*ApoE*	5′*-*CTTAAGAGCTCCACGCTTATC*-*3′	XM_051865683.1
	5′*-*GTGTAGTAGGACGCACATTTAT*-*3′	
*IL-10*	5′*-* AATCCCTTTGATTTTGCC*-*3′	XM_051913375.1
	5′*-*GTGCCTTATCCTACAGTATGTG*-*3′	
*CPT-1*	5′*-*AATTCTGCTTGACTTATGAG*-*3′	XM_051898996.1
	5′*-*CCTGTCCAAGGTACTTAGAC*-*3′	
*PPAR-*γ	5′*-*CGCTCATCTCCTACGGTCAG*-*3′	XM_051913344.1
	5′*-*ATGTCGCTGTCGTCCAACTC*-*3′	
*LPL*	5′*-*AGTACGCAGATGCCCAAAG*-*3′	XM_051909470.1
	5′*-*CTGGCCTCTGAATCCCAATAC*-*3′	

### DNA extraction, 16S rDNA amplification, and Illumina high throughput sequencing

The total bacterial DNA was extracted using the TGuide S96 magnetic bead method for soil/fecal genome DNA (Tiangen Biotech, Beijing, China), following the protocol provided by the manufacturer. The purity and concentration of genomic DNA were determined using the Qubit dsDNA HS Assay Kit and Qubit 4.0 Fluorometer (Invitrogen, Thermo Fisher Scientific, Oregon, United States). The extracted DNA was preserved at −80°C. The V3-V4 hypervariable region of the bacterial 16S rDNA gene was amplified using the primers 338F (5′−ACT CCT ACG GGA GGC AGC A−3′) and 806R (5′−GGA CTA CHV GGG TWT CTA AT−3′) (Mori et al., 2013). The PCR amplification program was the same as previously reported (Fu et al., 2019). PCR products were purified with Agencourt AMPure XP Beads (Beckman Coulter, Indianapolis, IN) and quantified with the Qubit dsDNA HS Assay Kit and Qubit 4.0 Fluorometer. Following individual quantification, equimolar amounts of amplicons were combined into a single pool. Sequencing was conducted by Biomarker technologies (Qingdao, China) on the Illumina NovaSeq 6000 sequencing platform. The raw 16S rRNA sequence data can be obtained in the NCBI SRA database (Bioproject: PRJNA1045724).

### Bioinformatics analysis of sequence data

The raw sequencing data were analyzed using the QIIME2 Pipeline, version 2021.4.^1^ The raw data were initially processed using Trimmomatic 0.35 (Bolger et al., 2014) for quality filtering, followed by the identification and removal of primer sequences with Cutadapt version 1.9.1 (Martin, 2011). DADA2 was subsequently employed to correct errors in the merged reads and to identify amplicon sequence variants (ASVs) (Callahan et al., 2016). The ASVs were then classified taxonomically using a native Bayes classifier (Wang et al., 2007), pre-trained on the SILVA 138 (Balvočiūtė and Huson, 2017).

Alpha diversity (ACE, Chao1, Simpson and Shannon index) of gut microbiota was calculated by QIIME2 and visualized in software R version 3.5. Beta diversity was used to evaluate the similarity between microbial communities across different samples in QIIME2. PERMANOVA analysis, non-metric multidimensional scaling (NMDS), Principal coordinate analysis (PCoA) and unweighted pair group mean algorithm (UPGMA) were used to visualize the beta diversity based on weighted Unifrac or Bray-curtis distance. The Pearson correlation coefficient was conducted using software PAST version 4.13 to examine the linear correlation between helminth infection status and the abundance of bacteria. Differences in taxa among groups were tested with Venn diagram and linear discriminant analysis coupled with effect size (Lefse). The metagenomic information of the samples was predicted from the 16S rDNA gene sequence data by employing PICRUST 2.0 in conjunction with the KEGG database (Langille et al., 2013). STAMP version 2.1.3 was used to perform all statistical analyses on the functional profiles (Parks et al., 2014).

### Statistical analysis

Data analysis among the four groups utilized the One-Way ANOVA, supplemented by LSD *post hoc* testing. Students’ *t*-test was used to perform statistically analysis between two groups. All the statistical tests were performed in software SPSS 20 at the 0.05 significance threshold.

## Results

### Role of helminth infection in the effect of HFD on growth indices of grass carp

**FIGURE 1:**
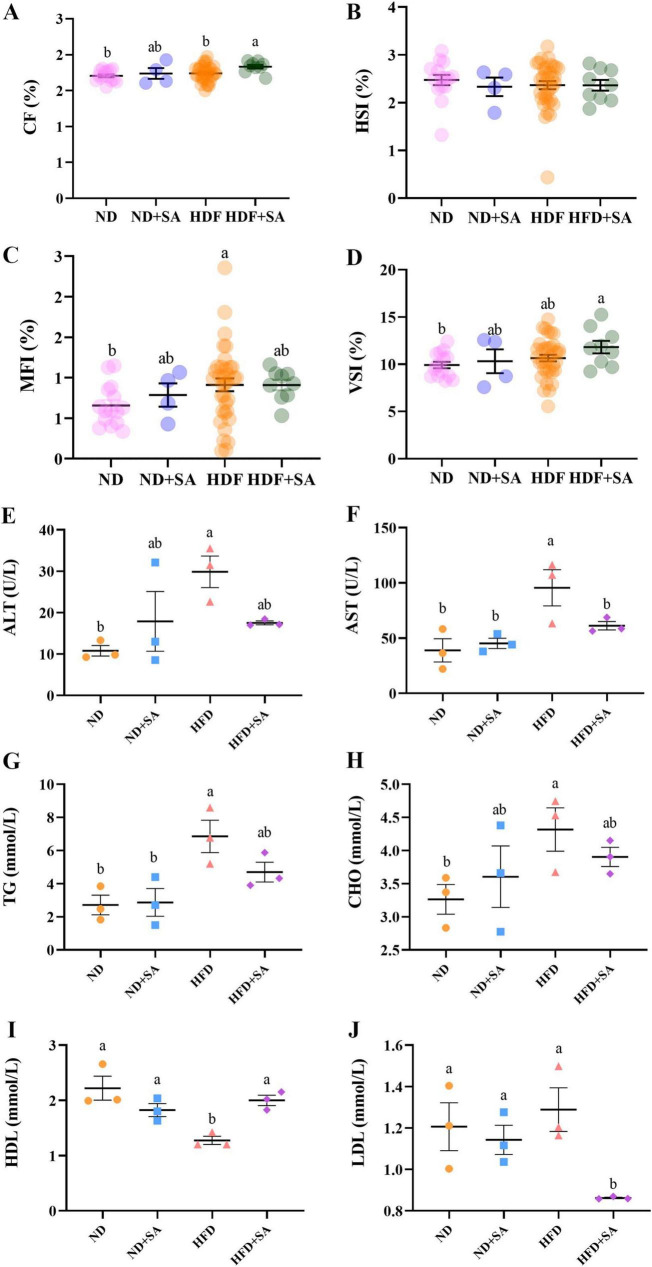
Effect of *S. acheilognathi* infection on growth performance and serum lipid parameters of *C. idella* fed on high-fat diet (HFD). **(A)** CF, condition factor; **(B)** HSI, hepatosomatic index; **(C)** VSI, visceral index; **(D)** MFI, mesenteric fat index; **(E)** ALT, alanine aminotransferase; **(F)** AST, aspartate aminotransferase; **(G)** TG, total triglycerides; **(H)** TC, total cholesterol; **(I)** HDL, high-density lipoprotein cholesterol **(J)** LDL, low-density lipoprotein cholesterol. **(A–D)** Values were presented as mean ± SEM (ND: *n* = 15; ND + SA: *n* = 4; HFD: *n* = 35; HFD + SA: *n* = 9); **(E–J)** values were presented as mean ± SEM (*n* = 3). ^a, b^Significant differences are indicated by different letters (*P* < 0.05). ND, 5% normal fat diet with no *S. acheilognathi* infection; ND + SA, 5% normal fat diet with *S. acheilognathi* infection; HFD, 10% high fat diet with no *S. acheilognathi* infection; HFD + SA, 10% high fat diet with *S. acheilognathi* infection.

The growth indices of grass carp were presented in Figures 1A–D. Among the four groups, HFD + SA had the highest CF, MFI and VSI (Figures 1A,C,D). The CF of HFD + SA group was significantly higher than that of the HFD group (*P* = 0.015 < 0.05) and ND group (*P* = 0.004 < 0.05) (Figure 1A); the MFI of HFD group was merely higher than that of ND group (*P* = 0.039 < 0.05, Figure 1C); and the VSI was only showed significant difference between the HFD + SA group and the ND group (*P* = 0.025 < 0.05, Figure 1D). The HSI did not exhibit any significant differences across the four groups (*P* > 0.05, in all cases, Figure 1B).

### Role of helminth infection in the effect of HFD on serum biochemical indices

The serum biochemical indices of grass carp were shown in Figures 1E–J. HFD significantly increased the activity of ALT (Figure 1E, *P* = 0.012) and AST (Figure 1F, *P* = 0.004), elevated serum levels of TG (Figure 1G, *P* = 0.005) and CHO (Figure 1H, *P* = 0.045), and reduced serum concentration of HDL (Figure 1I, *P* = 0.001) in comparison with normal diet. but the concentration of LDL between ND and HFD showed no significance (Figure 1J, *P* > 0.05). Comparing to HFD, helminth infection significantly reduced the activity of AST (Figure 1F, *P* = 0.044), decreased the concentration of LDL (Figure 1J, *P* = 0.008), and elevated the serum HDL content (Figure 1I, *P* = 0.006) in grass carp fed with HFD. The activity of ALT and the levels of TG and CHO in the serum showed a slight decrease in the HFD + SA group compared to the HFD group, but the differences were not significant (Figures 1E,G,H, *P* > 0.05 in all cases).

### Role of helminth infection in the effect of HFD on intestinal structure and liver lipid content

**FIGURE 2:**
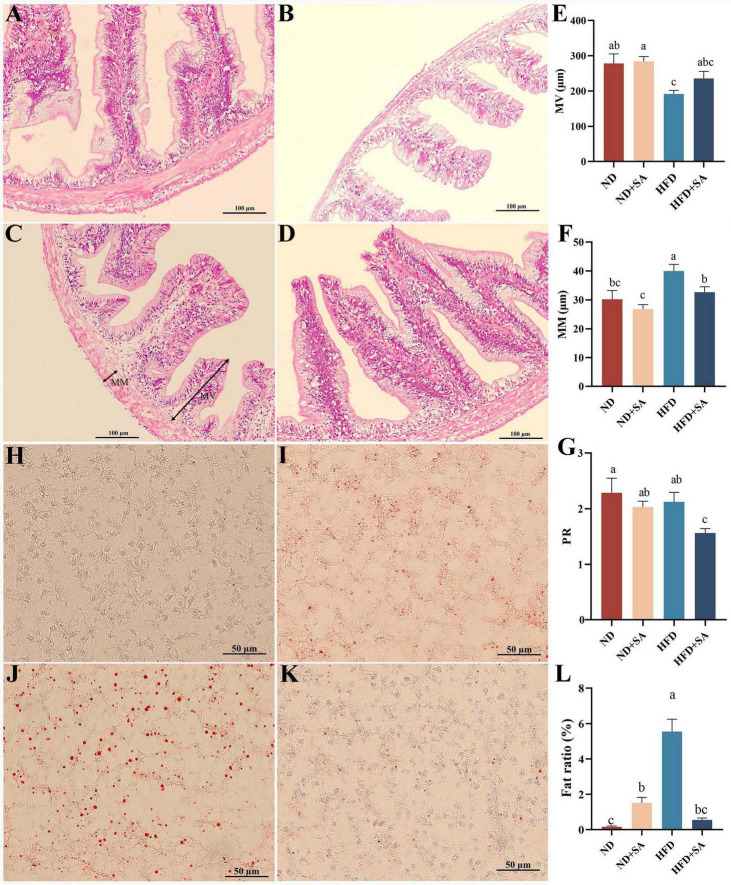
Effect of *S. acheilognathi* infection on intestinal morphology and liver lipid deposition of *C. idella* fed on HFD. **(A–D)** HE staining of intestine; **(H–K)** oil red O staining of liver. **(A,H)** ND; **(B,I)** ND + SA; **(C,J)** HFD; **(D,K)** HFD + SA; **(E)** MV, the height of microvilli; **(F)** MM, the thickness of muscularis mucosa; **(G)** PR, perimeter ratio; **(L)** Fat ratio. **(E–G,L)** Values were represented as mean ± SEM, **(E)** ND, *n* = 18; ND + SA, *n* = 23; HFD, *n* = 22; HFD + SA, *n* = 22; F: ND, *n* = 17; ND + SA, *n* = 24; HFD, *n* = 24; HFD + SA, *n* = 24; G: ND, *n* = 3; ND + SA, *n* = 4; HFD, *n* = 4; HFD + SA, *n* = 4; L: *n* = 5 for each group. ^a,b,c^Significant differences are indicated by different letters (*P* < 0.05). ND, 5% normal fat diet with no *S. acheilognathi* infection; ND + SA, 5% normal fat diet with *S. acheilognathi* infection; HFD, 10% high fat diet with no *S. acheilognathi* infection; HFD + SA, 10% high fat diet with *S. acheilognathi* infection.

The results of the hindgut sections stained with H&E were presented in Figures 2A–D. Helminth infection significantly decreased the MM (Figure 2F, *P* = 0.015) and PR (Figure 2G, *P* = 0.022) in grass carp fed on HFD, with no effect on MV (Figure 2E, *P* > 0.05). In the normal diet, helminth infection had no affection on the MM, MV, and PR (Figures 2E–G, *P* > 0.05 in all cases).

The liver lipid content was determined using oil red O staining. The results indicated that the lipid droplets in the HFD group were larger and denser comparing with the other three groups (Figures 2H–K). Helminth infection significantly decreased the fat ratio value in liver of grass carp fed with HFD (*P* = 0.000, Figure 2L). However, helminth infection significantly increased the fat ratio value in grass carp fed normal diet (*P* = 0.025, Figure 2L).

### Role of helminth infection in the effect of HFD on relative expression of immune and lipid metabolism related genes

**FIGURE 3:**
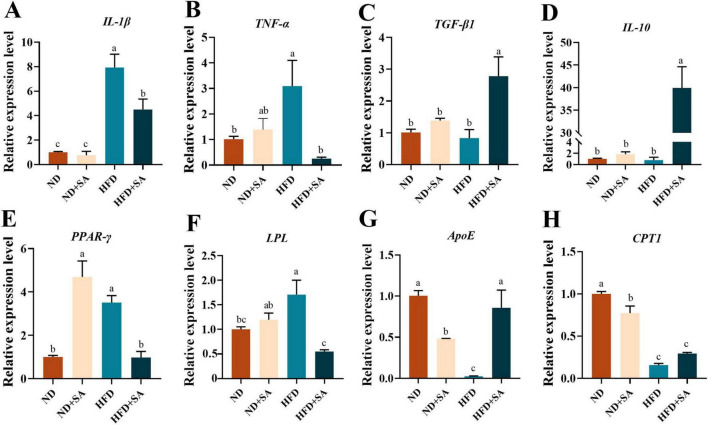
Effect of *S. acheilognathi* infection on the mRNA expression of immune and lipid metabolism related genes in *C. idella* fed on HFD. **(A)** IL-1β, interleukin-1beta; **(B)** TNF-α, tumor necrosis factor-alpha; **(C)** TGF-β1, transforming growth factor-beta 1; **(D)** IL-10, interleukin-10; **(E)** PPAR-γ, peroxisome proliferator-activated receptor-gamma; **(F)** LPL, lipoprotein lipase; **(G)** ApoE, apolipoprotein E; **(H)** CPT1, carnitine palmitoyltransferase 1; **(A–H)** Values were represented as mean ± SEM (*n* = 3); ^a,b,c^significant differences are indicated by different letters (*P* < 0.05). ND, 5% normal fat diet with no *S. acheilognathi* infection; ND + SA, 5% normal fat diet with *S. acheilognathi* infection; HFD, 10% high fat diet with no *S. acheilognathi* infection; HFD + SA, 10% high fat diet with *S. acheilognathi* infection.

The mRNA expression levels of immune and lipid metabolism related genes were showed in Figure 3. Helminth infection significantly promoted the expression of transforming growth factor-beta 1 (*TGF-*β*1*) (Figure 3C, *P* = 0.004), interleukin-10 (*IL-10*) (Figure 3D, *P* = 0.000) and apolipoprotein E (*ApoE*) (Figure 3G, *P* = 0.001), and significantly down-regulated the expression of interleukin-1beta (*IL-1*β) (Figure 3A, *P* = 0.009), tumor necrosis factor-alpha (*TNF-*α) (Figure 3B, *P* = 0.027), peroxisome proliferator-activated receptor-gamma (*PPAR-*γ) (Figure 3E, *P* = 0.003) and lipoprotein lipase (*LPL*) (Figure 3F, *P* = 0.001) in grass carp fed on HFD, but no affection on the expression of carnitine palmitoyltransferase 1(*CPT1*) (Figure 3H, *P* > 0.05). Meanwhile, helminth infection significantly elevated the relative expression levels of *PPAR-*γ (Figure 3E, *P* = 0.000), and significantly reduced the expression levels of *ApoE* (Figure 3G, *P* = 0.011) and *CPT1* (Figure 3H, *P* = 0.007), but no impact on the expression levels of *IL-1*β, *TNF-*α, *TGF-*β*1*, *IL-10*, and *LPL* (Figures 3A–D,F; *P* > 0.05 in all cases) in grass carp fed with normal diet.

### Role of helminth infection in the effect of HFD on the intestinal microbiota

#### Composition of intestinal microbiota

**FIGURE 4:**
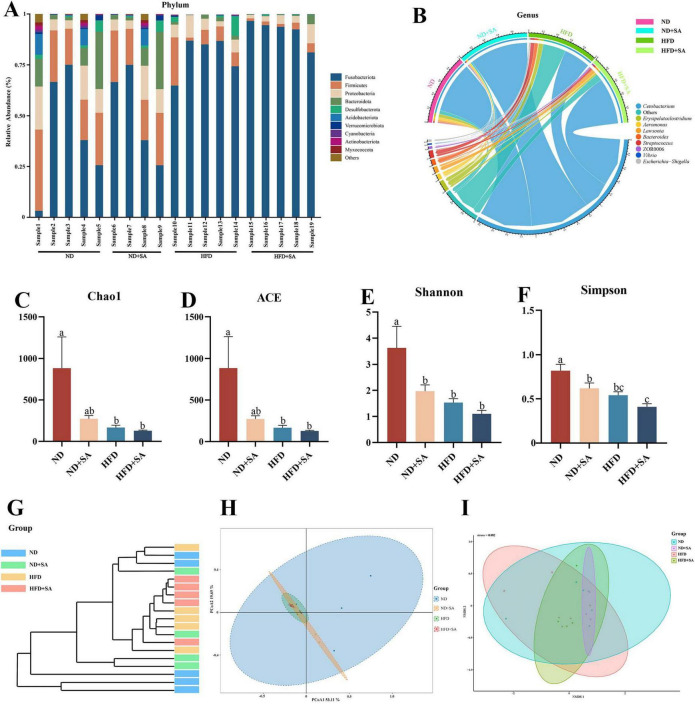
Effect of *S. acheilognathi* infection on the composition and diversity of intestinal microbiota in *C. idella* fed on HFD. **(A,B)** Microbiota composition; **(C–F)** α diversity indices; G-I: β diversity; **(A)** phylum level; **(B)** genus level; **(G)** cluster analysis; **(H)** PCoA, principal coordinates analysis; **(I)** NMDS, Non-metric multidimensional scaling; **(A–I)** ND, *n* = 5; ND + SA, *n* = 4; HFD, *n* = 5; HFD + SA, *n* = 5. ^a,b,c^Significant differences are indicated by different letters (*P* < 0.05). ND, 5% normal fat diet with no *S. acheilognathi* infection; ND + SA, 5% normal fat diet with *S. acheilognathi* infection; HFD, 10% high fat diet with no *S. acheilognathi* infection; HFD + SA, 10% high fat diet with *S. acheilognathi* infection.

The composition of intestinal microbiota was altered by helminth infection regardless of normal diet or high fat diet. At the phylum level (Figure 4A), the dominant taxa of the hindgut of grass carp were Fusobacteriota, Firmicutes, Proteobacteria and Bacteroidetes. Compared with the ND group, the relative abundance of Fusobacteria (72.59 ± 13.90% vs. 41.65 ± 29.51%) in the ND + SA was significantly increased (*P* = 0.019 < 0.05), and the relative abundance of Firmicutes (13.35 ± 6.73% vs. 25.75 ± 8.7%, *P* = 0.018 < 0.05), Proteobacteria (5.02 ± 2.61% vs. 11.84 ± 7.27%, *P* = 0.04 < 0.05) and Bacteroidetes (2.11 ± 1.06% vs. 10.82 ± 10.88%, *P* = 0.039 < 0.05) was significantly decreased. However, there was no significant difference in the relative abundance of dominant phyla of gut microbiota group between HFD and HFD + SA group in grass carp. Compared with HFD, the relative abundance of Fusobacteriota (91.70 ± 6.10% vs. 79.83 ± 9.72%) increased but not significant (*P* = 0.30 > 0.05) in HFD + SA group, and the relative abundance of Firmicutes (2.45 ± 1.40% vs. 9.24 ± 8.38%), Proteobacteria (4.27 ± 3.02% vs. 6.32 ± 3.06%), Bacteroidetes (1.12 ± 1.97% vs. 1.44 ± 0.78%), and Desulfobacterota (0.26 ± 0.26% vs. 2.62 ± 4.02%) in the HFD + SA group was also lower, but differences were not significant (*P* > 0.05 in all cases).

At the genus level, *Cetobacterium* was the dominant taxa in the hindgut of grass carp (Figure 4B). Compared with ND, the relative abundance of *Cetobacterium* (72.58 ± 13.91% vs. 41.46 ± 29.48%, *P* = 0.018 < 0.05, Figure 4B) in ND + SA was significantly increased, the relative abundance of *Aeromonas*, *Lawsonia*, and *ZOR0006* slightly increased (*P* > 0.05 in all cases, Figure 4B), and slightly decreased the relative abundance of *Streptococcus*, *Bacteroides* and *Erysipelatoclostridium* (*P* > 0.05 in all cases, Figure 4B). However, the relative abundance of *Cetobacterium* (91.58 ± 6.17% vs. 79.65 ± 9.86%, *P* = 0.30 > 0.05, Figure 4B) in HFD + SA group increased and the relative abundance of *Lawsonia*, *Bacteroides*, *Aeromonas*, and *Erysipelatoclostridium* all decreased comparing with HFD, but the differences were not significant (*P* > 0.05 in all cases, Figure 4B).

#### Diversity of gut microbiota

High fat diet significantly decreased the alpha diversity of hindgut microbiota in grass carp in comparison with normal diet (Figures 4C–F, *P* < 0.05 in all cases). However, helminth infection did not affect the alpha diversity of hindgut microbiota in grass carp fed on HFD (Figures 4C–F, *P* > 0.05 in all cases). Compared with HFD group, the HFD + SA group displayed a lower α diversity, but the differences were not significant (Figures 4C–F, *P* > 0.05 in all cases).

For the beta diversity, cluster analysis indicated that all samples were divided into two groups (Figure 4G), where almost all intestinal microbiota samples of grass carp fed on HFD clustered into one group, and samples that of feeding on ND clustered into a separate group. PERMANOVA (Table 3) analyses showed that the microbial communities of HFD + SA group was significantly different from HFD (*P* = 0.016). However, helminth infection had no affection on the microbial communities of grass carp fed normal diet (*P* = 0.064 > 0.05). The analyses of Principal coordinate analysis (PCoA) (Figure 4H) and non-metric multidimensional scaling (NMDS) (Figure 4I) showed that the microbial communities of the four groups could not be significantly distinguished from each other.

**TABLE 3:** PERMANOVA analysis of different groups with the Bray-Curtis distance.

Group	ND	ND + SA	HFD	HFD + SA
ND		3.255	1.529	**3.663**
ND + SA	0.064		**4.478**	0.659
HFD	0.192	***0.018***		**4.455**
HFD + SA	***0.010***	0.723	***0.016***	

Pseudo-F-values from the PERMANOVA test are displayed in standard font, *P*-values are presented in italics, and *P*-values < 0.05 are highlighted in bold.

#### Differences in taxonomic abundance among groups

**FIGURE 5:**
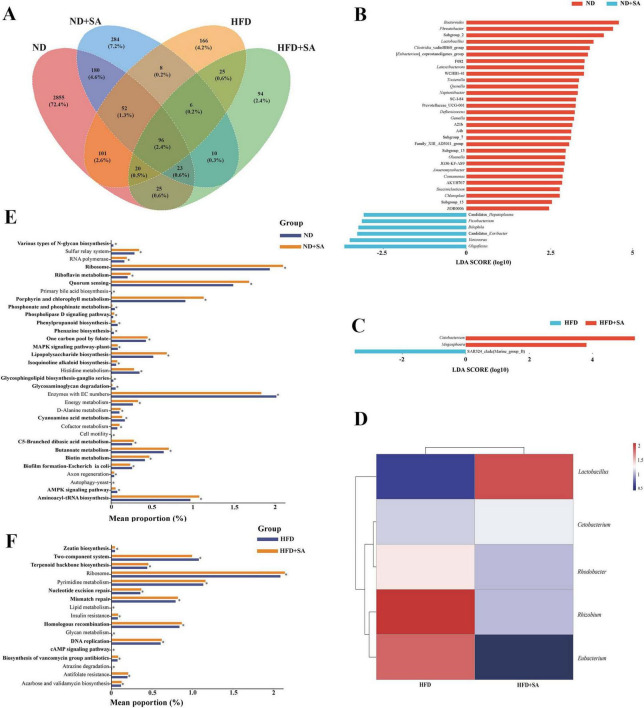
Differences and function of intestinal microbiota in *C. idella* fed on HFD. **(A)** Venn plot. **(B)** Lefse analysis between ND and ND + SA. **(C)** Lefse analysis between HFD and HFD + SA. **(D)** Heatmap of significant taxa in the intestine of HFD and HFD + SA. **(E,F)** Changes in the KEGG pathways predicted by PICRUST. **(E)** ND and ND + SA; **(F)** HFD and HFD + SA. **(A–F)** ND, *n* = 5; ND + SA, *n* = 4; HFD, *n* = 5; HFD + SA, *n* = 5. 0.01 < *P* < 0.05 values are marked with “*.” ND, 5% normal fat diet with no *S. acheilognathi* infection; ND + SA, 5% normal fat diet with *S. acheilognathi* infection; HFD, 10% high fat diet with no *S. acheilognathi* infection; HFD + SA, 10% high fat diet with *S. acheilognathi* infection.

A total of 3,968 ASVs were obtained in this study. Venn plot showed that total ASVs of ND, ND + SA, HFD and HFD + SA groups were 3,352, 659, 474, and 299, respectively; unique ASVs in four groups were 2,855, 284, 166, and 94, respectively; and the four groups shared 96 ASVs (Figure 5A).

Lefse analysis at the genus level indicated that there were thirty-six biomarkers between ND and ND + SA groups (Figure 5B), and only three biomarkers between HFD and HFD + SA groups (Figure 5C), including *Cetobacterium*, *Megasphaera*, and *SAR324_clade*.

#### Association between helminth infection and relative abundance microbiota in grass carp fed on HFD

Pearson correlation analysis indicated that helminth infection had a significant positive correlation with the relative abundance of *Cetobacterium* (*P* = 0.042, *r* = 0.65) and *Lactobacillus* (*P* = 0.001, *r* = 0.86), and three taxa, including *Rhodobacter*, *Rhizobium*, and *Eubacterium*, existed a significant negative correlation with the helminth infection (*P* < 0.05 in all cases; 0.63 ≤ |*r*| ≤ 0.71) (Figure 5D) in hindgut of grass carp fed on HFD (Table 4).

**TABLE 4:** Analysis of correlation coefficient between helminth infection and the taxonomic abundance of microbiota within the intestine of grass carp fed with HFD.

Taxon	*P*	*r*
*Lactobacillus*	0.001	0.86
*Cetobacterium*	0.042	0.65
*Rhodobacter*	0.019	−0.72
*Rhizobium*	0.033	−0.67
*Eubacterium*	0.046	−0.64

#### Functional alteration of gut microbiota

The PICRUST2 prediction identified thirty-three KEGG pathways at the L3 level with significant differences between the ND and ND + SA groups. Among these, twenty pathways pertained to metabolic processes, accounting for 60.6% (20/33), of which one pathway was associated with lipid metabolism, two with carbohydrate metabolism and one with energy metabolism (Figure 5E). In the comparison between HFD and HFD + SA, seventeen pathways exhibited notable differences. Of these, nine pathways were linked to metabolism functions (52.9%: 9/17), of which one KEGG pathway was related to lipid metabolism (Figure 5F).

## Discussion

The protective effect of helminth on metabolic diseases had been reported in mammals including improved insulin resistance, enhancing glucose tolerance, reducing blood lipids, inhibiting proinflammatory response and M2 macrophage proliferation etc. (Kang et al., 2021; Obi et al., 2020; Rajamanickam et al., 2020; Shimokawa et al., 2019; Su et al., 2020; Su et al., 2018; Yang et al., 2013), but the existence of such a protective effect in aquatic fauna remains unknown. In this study, we attempted to explore the role of helminth on metabolic diseases in aquatic animals. From our experimental results, the role of helminth in metabolic diseases in aquatic animals was similar to that in mammals. Helminth infection effectively ameliorated the high*-*fat diet induced metabolic imbalance in grass carp.

### The effect of *S. acheilognathi* infection on growth of grass carp fed on HFD

In growth performance, helminth infection with grass carp fed on HFD pointedly increased the CF, and slightly increased VSI and MFI. Numerous investigations on fish species, including *Micropterus salmoides* (Guo et al., 2019; Yin et al., 2021), *Nibea japonica* (Han et al., 2014), and *Rachycentron canadum* (Wang et al., 2005), have revealed a positive correlation between the VSI and MFI and the level of dietary lipids. The rise in CF observed in this study was associated with a concurrent upward trend in both VSI and MFI. The level of dietary fat usually causes no change of CF in fish (Guo et al., 2022; Han et al., 2014; Yin et al., 2021). Thus, the significant increasing of CF in HFD + SA group indicated that helminth infection boosted the growth of grass carp fed with HFD.

### The effect of *S. acheilognathi* infection on serum biochemical indices of grass carp fed on HFD

Helminth infection with grass carp fed with HFD exhibited a notable rise in LDL level, while AST level and HDL concentration significantly declined. AST is primarily situated within hepatocytes, and an increase in its serum level is a response to hepatocellular damage and changes in plasma membrane permeability (Boone et al., 2005). The high-fat diet led to an elevation in serum AST level in grass carp, indicating potential damage to liver cells. However, helminth infection could possibly decrease the liver’s burden imposed by the high-fat diet and facilitate the recovery of liver function. HDL and LDL are typically used as indicators to reflect lipid metabolism in aquatic animals (Guo et al., 2019; Li et al., 2016; Zhao et al., 2016) and mammals (Lu et al., 2022; Teng et al., 2020). High serum LDL is always considered to be risk factors related to fatty livers in fish (Guo et al., 2019; Li et al., 2016; Zhao et al., 2016). HDL has been referred to as good cholesterol, inversely proportional to cardiovascular risk in many studies (Ertek, 2018). In present study, the increase in HDL and decrease in LDL indicated that helminth infection reversed the abnormal lipid metabolism in HFD of grass carp, which could improve health condition of cultured *C. idella*.

### The effect of *S. acheilognathi* infection on lipid metabolism of grass carp fed on HFD

*PPAR-*γ, *LPL*, *ApoE*, and *CPT1* are the key regulatory enzymes involved in lipid metabolism within the liver, playing roles in the uptake, transport and oxidation of fatty acids. PPAR-γ is a ligand-activated transcription factor that belongs to the nuclear hormone receptor superfamily and fosters lipogenesis by enhancing the expression of enzymes involved in lipid synthesis (Ahmadian et al., 2013). LPL catalyzes the hydrolysis of intravascular triglycerides contained within lipoproteins, such as chylomicrons and very low-density lipoprotein (VLDL), then the released fatty acids can be taken up by tissues for use in oxidation or for storage purposes (Wu et al., 2021). ApoE is a soluble apolipoprotein primarily synthesized in the liver and brain, and it has an important role in the metabolism of triglyceride rich lipoproteins (TRL) due to its high affinity binding to the LDL receptor (LDLR), the LDL receptor-related protein 1 (LRP1) and the VLDL receptor (VLDLR), which facilitates the hepatic clearance of remnant lipoprotein particles (Huang and Mahley, 2014). LPL-mediated triglyceride hydrolysis would reduce when ApoE concentration was high (Wu et al., 2021). CPT1 is regarded as a pivotal regulatory enzyme in mitochondrial fatty acid oxidation pathway. It catalyzes the conversion of fatty acyl-CoAs into fatty acyl-carnitine molecules, which are then transported into the mitochondrial matrix for further oxidation (Kerner and Hoppel, 2000). In the present study, helminth infection in grass carp fed with HFD resulted in a modest elevation of *CPT1* expression, and caused a significantly reduction in the expression levels of *PPAR-*γ and *LPL*, while significantly increased the expression level of *ApoE*. These results indicated that helminth mainly regulate the expression of these lipid metabolism genes to inhibit the absorption of fatty acids, thereby reducing the deposition of lipid in the liver.

Additionally, the lipid content in the liver of the HFD + SA group was significantly lower than that of the HFD group, indicating that helminth infection mitigated the liver lipid deposition induced by the high-fat diet. The protective effect of helminth in grass carp was similar to that in mice (Su et al., 2018). Most of the digested lipids are absorbed by intestinal epithelial cells (Zhang et al., 2021). PR, one of the intestinal morphology parameters, was significantly decreased by helminth infection in this study, reducing the amount of lipid absorbed by intestine, thereby to decrease lipid deposition in the liver.

### The effect of *S. acheilognathi* infection on immune response and gut microbiota of grass carp fed on HFD

Similarity to the protective effects of helminth in mammalian metabolic diseases, the primary regulatory pathways through which helminths exert positive effects on metabolic diseases in fish may include the following two aspects: (1) immune regulation, (2) alterations of gut microbiota. The type 2 immune response induced by helminth infection has been proved to ameliorate the side effects of HFD in mice (Su et al., 2020; Su et al., 2018). The intestinal nematode *H. polygyrus* could protect against obesity by triggering the production of Th2-mediated cytokines, such as IL-4, lL-10 and IL-13, and enhancing the anti-inflammatory M2 macrophages levels (Su et al., 2018). The increased M2 and IL-10 responses resulted in a reduction of the pro-inflammatory cytokine TNF-α expression, which has been shown to modulate lipid metabolism (Khovidhunkit et al., 2004). Similarly, in our study, helminth infection significantly elevated IL-10 levels while concurrently reducing decreased TNF-α expression, indicating that helminth infection protects against the HFD-induced abnormal lipid metabolism via activation of immune responses in teleost.

An increase of alpha diversity in intestinal microbiota is generally associated with a “healthy” gut homeostasis (Peachey et al., 2017). In this study, a high-fat diet significantly decreased the α diversity of intestinal microbiota in grass carp (Figures 4C–F), indicating that HFD disrupted gut microbial balance. Similar disruptions caused by HFD have also been reported in *Monopterus albus* (Peng et al., 2019). Additionally, there was no significant difference in the α diversity of gut microbiota between the HFD group and the HFD + SA group (Figures 4C–F), which may be attributed to the regulatory effect of helminth on intestine microbiota.

Microbiota and helminths occupy the same ecological niche within the host’s intestine, where they can interact with each other (Glendinning et al., 2014). Multiple investigations have been carried out to examine the effects of helminth infection on the gut microbiota in fish. Tapeworm and acanthocephalan infection caused alteration in the composition of fish gut microbiota (Fu et al., 2019; Fu et al., 2022; Jiang et al., 2019; Ling et al., 2020). Studies in mammals have shown that helminth infection protects against HFD-induced obesity through modifications to the gut microbiota (Shimokawa et al., 2019; Su et al., 2020).

helminth infection was found to enhance the relative abundance of *Cetobacterium* in hindgut of grass carp fed with HFD, and the proportion of *Cetobacterium* in the HFD + SA group was 91.6%. According to Spearman’s correlation analysis, *Cetobacterium* correlated positively with helminth infection. *Cetobacterium*, identified as an anaerobic, gram-negative bacterium (Finegold et al., 2003), constitutes a predominant member of the intestinal microbiota in freshwater fish (Di Maiuta et al., 2013; Fu et al., 2022; Larsen et al., 2014; van Kessel et al., 2011). The significant proliferation of *Cetobacterium* could perform fermentative metabolism of peptides and carbohydrates, resulting in the production of acetate, which in turn modifies glucose homeostasis (Wang et al., 2021). Additionally, this bacterium could produce vitamin B12 (Larsen et al., 2014), thereby enhancing the host couldsicrobioto pathogen infections (Qi et al., 2023). Incorporating *Cetobacterium* fermentation products into fish feed can effectively enhance intestinal and liver health and decrease liver lipid accumulation (Xie et al., 2022). In zebrafish, cypermethrin (CYP) exposure or CYP and microplastics (MPs) co-exposure increased the abundance of *Cetobacterium*, which was found to be positively associated with the majority of lipid metabolites (Xu et al., 2024). Consistently, helminth infection may ameliorate the lipid metabolism induced by HFD through increasing the high relative abundance of *Cetobacterium* to regulate lipid and maintain liver health of grass carp.

*Lactobacillus*, a group of gram-positive, facultative anaerobic bacteria widely used as probiotics, has been shown to enhance epithelial barrier function and modulate innate immune responses as well as cytokine profiles (Amdekar and Singh, 2016). *Strongyloides venezuelensis* infection in mice induced the increasing of *Lactobacillus* spp., which has a positive effect on the glucose metabolism of the host (Pace et al., 2018). In this study, helminth infection showed a significant positive correlation with *Lactobacillus*. This result indicated that helminth infection can improve host metabolic profile by regulating probiotics.

In study of *Caenorhabditis elegans*, reactive oxygen species produced by *Rhizobium* induce DNA damage, which caused abnormal intestinal nuclei divisions (Kniazeva and Ruvkun, 2019). *Eubacterium*, one of the main SCFAs-producing bacteria in humans (Parada Venegas et al., 2019). *Rhodobacter* is a gram-negative, purple non-sulfur photosynthetic bacteria. Orally given the *Rhodobacter sphaeroides* in mice increased the content of SCFAs (Yang et al., 2020). However, SCFA has a significant negative influence on established *Oesophagostomum dentatum* infection in pigs (Petkevicius et al., 2004). In this study, helminth infection showed significant positive correlation with *Rhodobacter*, *Rhizobium*, and *Eubacterium*, indicating that helminth may improve its survival in the intestine by altering the relative abundance of these three bacteria.

## Conclusion

In conclusion, the findings of this study showed that helminth infection can ameliorate the abnormal lipid metabolism caused by a high-fat diet in grass carp. Helminth infection decreased the lipid deposition in liver, the concentration of LDL and activity of AST in serum, while increased the level of HDL. The synergistic effect of IL-10 produced by helminth mediated Th2 immune response and *Cetobacterium* alteration in intestine induced by helminth infection upregulated the expression level of *ApoE* and downregulated the expression level of *PPAR-*γ and *LPL* in liver of grass carp, thus improving lipid metabolism.

## Data Availability

The original contributions presented in the study are publicly available. This data can be found at: https://www.ncbi.nlm.nih.gov, accession number PRJNA1045724.

## Footnotes

1
https://docs.qiime2.org/
